# *rac*-1-(2-Amino­carbonyl-2-bromo­eth­yl)pyridinium bromide

**DOI:** 10.1107/S1600536812019721

**Published:** 2012-05-12

**Authors:** Robert Köppen, Franziska Emmerling, Matthias Koch

**Affiliations:** a BAM Federal Institute for Materials Research and Testing, Department of Analytical Chemistry, Reference Materials, Richard-Willstätter-Strasse 11, D-12489 Berlin-Adlershof, Germany

## Abstract

In the crystal structure of the title compound, C_8_H_10_BrN_2_O^+^·Br^−^, inter­molecular N—H⋯Br hydrogen bonds link the mol­ecules into infinite chains along [001]. The inclined angle between the pyridine ring plane and the plane defined by the acid amide group is 63.97 (4)°.

## Related literature

The title compound is an inter­mediate in the synthesis of 3-triphenyl­phospho­nium­bromidopropionitrile and 1-tri­phen­yl­phospho­nium­bromido-2-pyridinium-bromidoethane, see: Khach­ikyan *et al.* (2009 ▶).
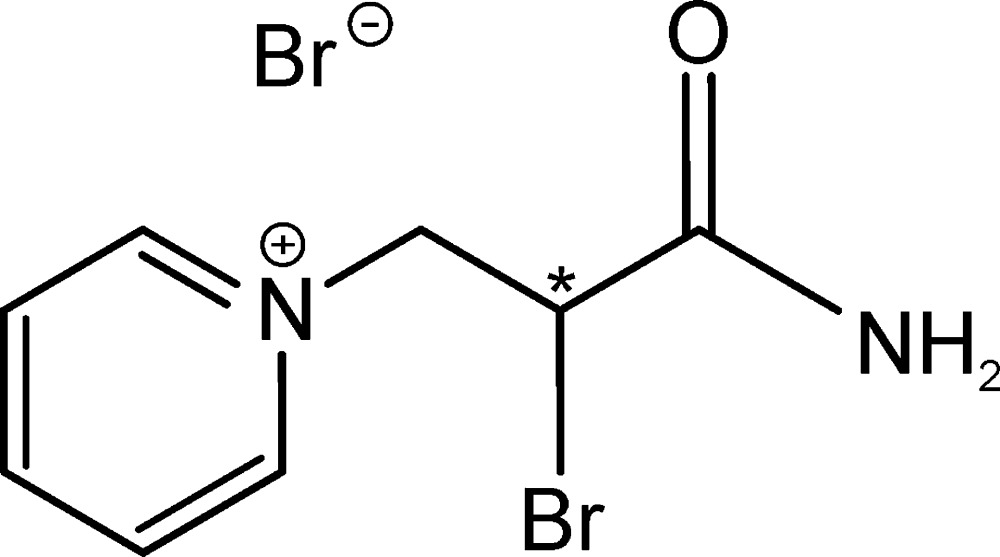


## Experimental

### 

#### Crystal data

C_8_H_10_BrN_2_O^+^·Br^−^
*M* *_r_* = 310.00
Monoclinic, 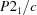

*a* = 8.6024 (9) Å
*b* = 16.1200 (19) Å
*c* = 9.5092 (12) Å
β = 121.501 (8)°
*V* = 1124.3 (2) Å^3^
*Z* = 4
Mo *K*α radiation
μ = 7.18 mm^−1^
*T* = 296 K
0.14 × 0.11 × 0.05 mm

#### Data collection

Bruker SMART APEX CCD area-detector diffractometer
Absorption correction: multi-scan (*SADABS*; Bruker, 2001 ▶) *T*_min_ = 0.85, *T*_max_ = 0.96
10331 measured reflections
2252 independent reflections
1442 reflections with *I* > 2σ(*I*)
*R*_int_ = 0.106

#### Refinement

*R*[*F* ^2^ > 2σ(*F* ^2^)] = 0.044
*wR*(*F* ^2^) = 0.129
*S* = 1.00
2252 reflections
118 parameters
H-atom parameters constrained
Δρ_max_ = 0.96 e Å^−3^
Δρ_min_ = −0.56 e Å^−3^

### 

Data collection: *SMART* (Bruker, 2001 ▶); cell refinement: *SAINT* (Bruker, 2001 ▶); data reduction: *SAINT*; program(s) used to solve structure: *SHELXS97* (Sheldrick, 2008 ▶); program(s) used to refine structure: *SHELXL97* (Sheldrick, 2008 ▶); molecular graphics: *SHELXTL* (Sheldrick, 2008) ▶ and *ORTEPIII* (Burnett & Johnson, 1996 ▶); software used to prepare material for publication: *SHELXTL*.

## Supplementary Material

Crystal structure: contains datablock(s) I, global. DOI: 10.1107/S1600536812019721/fj2549sup1.cif

Structure factors: contains datablock(s) I. DOI: 10.1107/S1600536812019721/fj2549Isup2.hkl

Supplementary material file. DOI: 10.1107/S1600536812019721/fj2549Isup3.mol

Supplementary material file. DOI: 10.1107/S1600536812019721/fj2549Isup4.cml

Additional supplementary materials:  crystallographic information; 3D view; checkCIF report

## Figures and Tables

**Table 1:** Hydrogen-bond geometry (Å, °)

*D*—H⋯*A*	*D*—H	H⋯*A*	*D*⋯*A*	*D*—H⋯*A*
N2—H2*A*⋯Br2^i^	0.86	2.62	3.406 (7)	154
N2—H2*B*⋯Br2^ii^	0.86	2.57	3.428 (6)	173

Symmetry codes: (i) 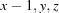; (ii) 
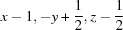
.

**Fig. 1.**
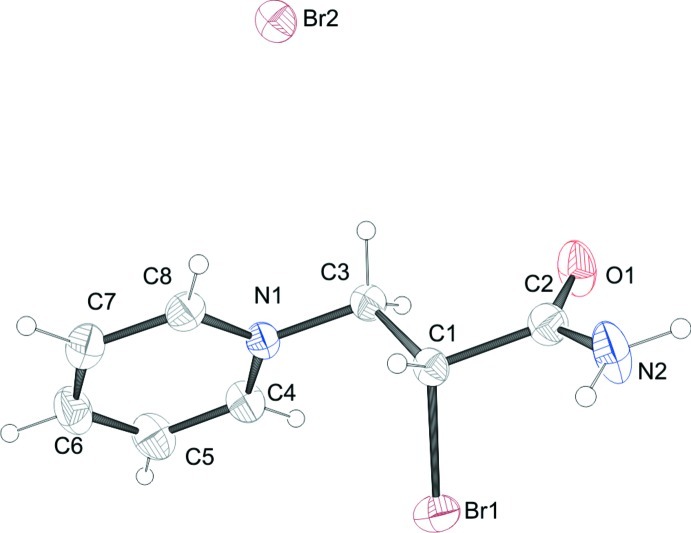
: ORTEP representation of the title compound with atomic labeling shown with 30% probability displacement ellipsoids.

**Fig. 2.**
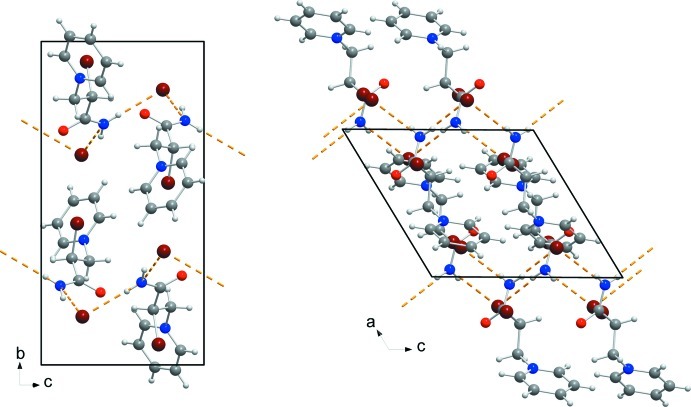
: View of the unit cell of the title compound along [100] (left) and [010] (right) showing the hydrogen-bonded chains along the [001] direction. Hydrogen bonds are drawn as dashed orange lines.

## supplementary crystallographic information

### Comment

*rac*-1-(2-Aminocarbonyl-2-bromoethyl)pyridinium bromide is an intermediate in the synthesis of 3-triphenylphosphoniumbromidopropionitrile and 1-triphenylphosphoniumbromido-2-pyridinium-bromidoethane (Khachikyan *et al.*, 2009). The compound crystallizes in the monoclinic space group *P*2_1_/c. The molecular structure of the compound and the atom-labeling scheme are shown in Fig 1. The molecules are arranged in such a way that the pyridyl rings are stagged with respect to each other. However, the distance between the molecular planes (d_centroids_=4.295 (4) Å) indicates only weak π-π interactions. Each molecule is connected to two adjacent bromine anions *via* intermolecular N—H···Br hydrogen bonds (see dashed orange bonds in Fig. 2). As a result infinite chains are formed along [001] direction.

### Experimental

In a 250 ml, one-necked, round-bottomed flask fitted with a reflux condenser and a magnetic stirrer a mixture of 1.85 g of rac-2,3-dibromopropionic acid amide (7.98 mmol) and 0.63 g of pyridine (7.98 mmol) was diluted in 100 ml of acetonitrile and refluxed for 25 h. After cooling the flask was capped with a rubber septum equipped with a needle outlet for slow evaporation and then left in the dark at room temperature for 3 days. The obtained colorless crystals were suitable for direct single-crystal X-ray crystallography.

### Refinement

All H-atoms were positioned geometrically and refined using a riding model with d(C—H) = 0.93 Å, *U*_iso_=1.2*U*_eq_ (C) for aromatic 0.98 Å, *U*_iso_ = 1.2*U*_eq_ (C) for CH, 0.97 Å, *U*_iso_ = 1.2*U*_eq_ (C) for CH_2_, 0.96 Å, *U*_iso_ = 1.5*U*_eq_ (C) for CH_3_ atoms, and 0.82 Å, *U*_iso_ = 1.5*U*_eq_ (C) for the amino group.

### Crystal data

**Table:**

C_8_H_10_BrN_2_O^+^·Br^−^	*F*(000) = 600
*M**_r_* = 310.00	*D*_x_ = 1.831 Mg m^−^^3^
Monoclinic, *P*2_1_/*c*	Mo *K*α radiation, λ = 0.71073 Å
Hall symbol: -P 2ybc	Cell parameters from 42 reflections
*a* = 8.6024 (9) Å	θ = 4–25°
*b* = 16.1200 (19) Å	µ = 7.18 mm^−^^1^
*c* = 9.5092 (12) Å	*T* = 296 K
β = 121.501 (8)°	Block, colourless
*V* = 1124.3 (2) Å^3^	0.14 × 0.11 × 0.05 mm
*Z* = 4	

### Data collection

**Table:**

Bruker SMART APEX CCD area-detector diffractometer	2252 independent reflections
Radiation source: fine-focus sealed tube	1442 reflections with *I* > 2σ(*I*)
Graphite monochromator	*R*_int_ = 0.106
ω/2θ scans	θ_max_ = 26.3°, θ_min_ = 2.5°
Absorption correction: multi-scan (*SADABS*; Bruker, 2001)	*h* = −10→10
*T*_min_ = 0.85, *T*_max_ = 0.96	*k* = −19→18
10331 measured reflections	*l* = −11→11

### Refinement

**Table:**

Refinement on *F*^2^	Primary atom site location: structure-invariant direct methods
Least-squares matrix: full	Secondary atom site location: difference Fourier map
*R*[*F*^2^ > 2σ(*F*^2^)] = 0.044	Hydrogen site location: inferred from neighbouring sites
*wR*(*F*^2^) = 0.129	H-atom parameters constrained
*S* = 1.00	*w* = 1/[σ^2^(*F*_o_^2^) + (0.0659*P*)^2^] where *P* = (*F*_o_^2^ + 2*F*_c_^2^)/3
2252 reflections	(Δ/σ)_max_ < 0.001
118 parameters	Δρ_max_ = 0.96 e Å^−^^3^
0 restraints	Δρ_min_ = −0.56 e Å^−^^3^

### Special details

**Table:**

Geometry. All e.s.d.'s (except the e.s.d. in the dihedral angle between two l.s. planes) are estimated using the full covariance matrix. The cell e.s.d.'s are taken into account individually in the estimation of e.s.d.'s in distances, angles and torsion angles; correlations between e.s.d.'s in cell parameters are only used when they are defined by crystal symmetry. An approximate (isotropic) treatment of cell e.s.d.'s is used for estimating e.s.d.'s involving l.s. planes.
Refinement. Refinement of *F*^2^ against ALL reflections. The weighted *R*-factor *wR* and goodness of fit *S* are based on *F*^2^, conventional *R*-factors *R* are based on *F*, with *F* set to zero for negative *F*^2^. The threshold expression of *F*^2^ > σ(*F*^2^) is used only for calculating *R*-factors(gt) *etc*. and is not relevant to the choice of reflections for refinement. *R*-factors based on *F*^2^ are statistically about twice as large as those based on *F*, and *R*- factors based on ALL data will be even larger.

### Fractional atomic coordinates and isotropic or equivalent isotropic displacement parameters (Å^2^)

**Table:**

	*x*	*y*	*z*	*U*_iso_*/*U*_eq_	
Br1	0.23648 (10)	0.06295 (5)	0.72065 (9)	0.0538 (3)	
O1	0.3105 (6)	0.2759 (3)	0.8640 (6)	0.0652 (15)	
N1	0.6197 (6)	0.1137 (3)	0.7616 (6)	0.0384 (12)	
N2	0.0498 (7)	0.2553 (4)	0.6168 (7)	0.0661 (18)	
H2A	−0.0084	0.2935	0.6335	0.079*	
H2B	−0.0038	0.2274	0.5263	0.079*	
C1	0.3180 (8)	0.1706 (4)	0.6882 (7)	0.0357 (14)	
H1	0.2813	0.1761	0.5722	0.043*	
C2	0.2227 (9)	0.2392 (4)	0.7308 (8)	0.0460 (16)	
C3	0.5231 (8)	0.1790 (4)	0.7954 (7)	0.0408 (15)	
H3A	0.5591	0.2329	0.7764	0.049*	
H3B	0.5590	0.1762	0.9104	0.049*	
C4	0.6778 (10)	0.0453 (4)	0.8562 (8)	0.0487 (17)	
H4	0.6589	0.0398	0.9435	0.058*	
C5	0.7646 (11)	−0.0161 (5)	0.8247 (9)	0.061 (2)	
H5	0.8030	−0.0639	0.8891	0.073*	
C6	0.7945 (10)	−0.0071 (5)	0.6982 (10)	0.061 (2)	
H6	0.8561	−0.0482	0.6778	0.073*	
C7	0.7336 (10)	0.0633 (4)	0.6000 (9)	0.0541 (18)	
H7	0.7516	0.0698	0.5123	0.065*	
C8	0.6458 (8)	0.1229 (4)	0.6362 (8)	0.0432 (15)	
H8	0.6038	0.1707	0.5720	0.052*	
Br2	0.79943 (9)	0.35088 (4)	0.75292 (7)	0.0465 (3)	

### Atomic displacement parameters (Å^2^)

**Table:**

	*U*^11^	*U*^22^	*U*^33^	*U*^12^	*U*^13^	*U*^23^
Br1	0.0548 (5)	0.0502 (5)	0.0573 (5)	−0.0118 (3)	0.0299 (4)	−0.0013 (3)
O1	0.047 (3)	0.087 (4)	0.050 (3)	0.002 (3)	0.018 (3)	−0.035 (3)
N1	0.038 (3)	0.039 (3)	0.038 (3)	0.002 (2)	0.020 (3)	−0.003 (2)
N2	0.045 (4)	0.079 (4)	0.054 (4)	0.019 (3)	0.012 (3)	−0.027 (3)
C1	0.044 (4)	0.038 (3)	0.027 (3)	0.001 (3)	0.020 (3)	−0.001 (2)
C2	0.049 (5)	0.050 (4)	0.041 (4)	−0.002 (3)	0.025 (4)	−0.005 (3)
C3	0.040 (4)	0.040 (4)	0.044 (3)	−0.001 (3)	0.022 (3)	−0.003 (3)
C4	0.059 (5)	0.046 (4)	0.041 (4)	0.008 (3)	0.026 (4)	0.006 (3)
C5	0.068 (5)	0.051 (5)	0.057 (4)	0.018 (4)	0.028 (4)	0.010 (4)
C6	0.045 (4)	0.064 (5)	0.067 (5)	0.010 (4)	0.024 (4)	−0.016 (4)
C7	0.051 (5)	0.064 (5)	0.053 (4)	0.001 (4)	0.032 (4)	−0.001 (4)
C8	0.039 (4)	0.047 (4)	0.043 (4)	0.000 (3)	0.020 (3)	0.004 (3)
Br2	0.0511 (5)	0.0474 (4)	0.0384 (4)	0.0015 (3)	0.0216 (3)	−0.0004 (3)

### Geometric parameters (Å, º)

**Table:**

Br1—C1	1.956 (6)	C3—H3A	0.9700
O1—C2	1.235 (7)	C3—H3B	0.9700
N1—C8	1.333 (7)	C4—C5	1.364 (9)
N1—C4	1.343 (8)	C4—H4	0.9300
N1—C3	1.477 (7)	C5—C6	1.363 (10)
N2—C2	1.330 (8)	C5—H5	0.9300
N2—H2A	0.8599	C6—C7	1.386 (10)
N2—H2B	0.8601	C6—H6	0.9300
C1—C3	1.513 (8)	C7—C8	1.373 (9)
C1—C2	1.550 (9)	C7—H7	0.9300
C1—H1	0.9800	C8—H8	0.9300
			
C8—N1—C4	120.9 (5)	N1—C3—H3B	109.1
C8—N1—C3	119.4 (5)	C1—C3—H3B	109.1
C4—N1—C3	119.7 (5)	H3A—C3—H3B	107.9
C2—N2—H2A	120.0	N1—C4—C5	120.3 (6)
C2—N2—H2B	120.0	N1—C4—H4	119.9
H2A—N2—H2B	120.0	C5—C4—H4	119.9
C3—C1—C2	110.5 (5)	C4—C5—C6	119.5 (7)
C3—C1—Br1	111.2 (4)	C4—C5—H5	120.2
C2—C1—Br1	108.0 (4)	C6—C5—H5	120.2
C3—C1—H1	109.1	C5—C6—C7	120.2 (7)
C2—C1—H1	109.1	C5—C6—H6	119.9
Br1—C1—H1	109.1	C7—C6—H6	119.9
O1—C2—N2	124.5 (6)	C8—C7—C6	117.9 (6)
O1—C2—C1	119.0 (6)	C8—C7—H7	121.1
N2—C2—C1	116.4 (5)	C6—C7—H7	121.1
N1—C3—C1	112.3 (5)	N1—C8—C7	121.2 (6)
N1—C3—H3A	109.1	N1—C8—H8	119.4
C1—C3—H3A	109.1	C7—C8—H8	119.4
			
C3—C1—C2—O1	−18.5 (8)	C8—N1—C4—C5	−0.2 (10)
Br1—C1—C2—O1	103.3 (6)	C3—N1—C4—C5	178.9 (6)
C3—C1—C2—N2	160.2 (6)	N1—C4—C5—C6	1.1 (11)
Br1—C1—C2—N2	−78.1 (6)	C4—C5—C6—C7	−1.5 (12)
C8—N1—C3—C1	83.1 (7)	C5—C6—C7—C8	1.0 (11)
C4—N1—C3—C1	−96.1 (7)	C4—N1—C8—C7	−0.3 (9)
C2—C1—C3—N1	178.7 (5)	C3—N1—C8—C7	−179.4 (6)
Br1—C1—C3—N1	58.8 (6)	C6—C7—C8—N1	−0.1 (10)

### Hydrogen-bond geometry (Å, º)

**Table:**

*D*—H···*A*	*D*—H	H···*A*	*D*···*A*	*D*—H···*A*
N2—H2*A*···Br2^i^	0.86	2.62	3.406 (7)	154
N2—H2*B*···Br2^ii^	0.86	2.57	3.428 (6)	173

Symmetry codes: (i) *x*−1, *y*, *z*; (ii) *x*−1, −*y*+1/2, *z*−1/2.
